# BAAV Transcytosis Requires an Interaction with β-1-4 Linked- Glucosamine and gp96

**DOI:** 10.1371/journal.pone.0009336

**Published:** 2010-03-09

**Authors:** Giovanni Di Pasquale, Nikola Kaludov, Mavis Agbandje-McKenna, John A. Chiorini

**Affiliations:** 1 Molecular Physiology and Therapeutics Branch, National Institute of Dental and Craniofacial Research, National Institutes of Health, Bethesda, Maryland, United States of America; 2 Department of Biochemistry and Molecular Biology, The McKnight Brain Institute, College of Medicine, University of Florida, Gainesville, Florida, United States of America; National Cancer Institute, United States of America

## Abstract

Cell surface carbohydrates play an important role in virus entry and intracellular trafficking. Bovine Adeno-Associated Virus (BAAV) uses plasma membrane gangliosides for transduction and infection. In addition, independent of the infectious pathway, BAAV also has the ability to pass through barrier epithelia and endothelia using a transcytosis pathway dependent upon the presence of cell surface carbohydrates. Thus, in order to better define the carbohydrate interactions that are necessary for BAAV infection or transcytosis, a glycan microarray composed of both natural and synthetic carbohydrates was probed with HA-tagged BAAV particles. This identified chitotriose, a trimer of β-1-4-linked N-acetyl glucosamine, as having an interaction with BAAV. Competition experiments showed that the BAAV interaction with this carbohydrate is not necessary for infection but is instead important in the transcytosis pathway. The β-1-4-linked N-acetyl glucosamine modification has been reported on gp96, a glycoprotein involved in the transcytosis of bacteria and toxins. Significantly, immunoprecipitation and competition experiments with an anti-gp96 antibody and a soluble form of gp96, respectively, showed this glycoprotein can also interact with BAAV to serve as a receptor for its transcytosis.

## Introduction

The initial interaction between a virus and the cell surface is critical for determining the fate of the virus. While some interactions will lead to entry and infection of the cells, others will lead to the destruction of the virus in the cytosol [1]–[3]. Carbohydrate interactions play an important role in the life cycle of viruses. In addition to serving as a point of attachment for the virus on the cell surface, carbohydrates on viral proteins are also important in intracellular trafficking [4].

Adeno-associated viruses (AAVs) have been isolated from a wide variety of species and are reported to bind a diverse array of cell surface carbohydrates including heparan sulfate proteoglycans, N- and O-linked sialyated glycans, and glycoshingolipids (reviewed in [5]). While the majority of interactions between AAV and carbohydrates have been associated with cell binding, virus entry, and infection, it is possible that they are also involved in other aspects of the virus lifecycle.

Previous work from our group with an AAV isolated from a stock of bovine adenovirus, termed bovine AAV (BAAV) [6] demonstrated that this virus did not require cell surface proteins for entry but instead used gangliosides for cell infection and transduction [7]. This tropism resulted in gene transfer vectors based on BAAV having a unique tropism compared with current AAV vectors and the ability to transduce cells in the inner ear [8]–[10]. In addition, this and other AAV serotypes, for example AAV4 and AAV5, have the ability to pass through barrier epithelia and endothelia using transcytosis via a pathway independent of that used for infection [11]. Our experiments with BAAV, AAV4, and AAV5 suggested that this process is rapid as well as serotype and cell-type specific and can be blocked by neutralizing antibodies, temperature, or chemical inhibitors of transcytosis. We observed that depending on the cell type and vector, as much as 3 to 15% of the vector that binds to the cell surface will transcytosis through a permissive barrier cell model over 24 hrs [11]. Furthermore, competition experiments with lectins suggested the involvement of cell surface carbohydrates in this process [11]. Interestingly, particles isolated following apical-to-basolateral transcytosis still have their genomes encapsulated and can transduce permissive cell lines *in vitro* [11].

Transcytosis has been reported for a number of macromolecules as well as pathogens such as bacteria and viruses. At its most basic level, transcytosis is the movement of macromolecules from one side of a cell to the other and can occur by a variety of mechanisms [12]. For HIV transcytosis, cell surface galactosyl-ceramides have been proposed as a receptor for this pathway [13]. For other pathogens such as *S. pneumoniae*, translocation across a barrier layer via the poly(IgA) receptor [14], [15] has been proposed. As described above, transcytosis activity has been demonstrated in BAAV, AAV4, AAV5 and has been proposed to occur in 2 others; AAV8 and AAV9 [11], [16]–[19]. However, little is known regarding the mechanism of AAV transcytosis. In order to better define the carbohydrate interactions that are necessary for BAAV transduction or transcytosis we probed a glycan microarray composed of carbohydrates commonly found on the cell surface with BAAV particles tagged with an antigenic peptide from the influenza hemagglutinin (HA) protein (HA-BAAV particles). This analysis identified an interaction with chitotriose, a trimer of β-1-4 linked N-acetyl glucosamine (GlcNAc). This interaction was specific for BAAV and was not seen with an HA-tagged AAV4, which has a high amino acid sequence homology to BAAV as well as a distinct but overlapping transduction and transcytosis activity [6], [11]. Competition experiments showed that the interaction of BAAV with this carbohydrate is not necessary for transduction but is important in the transcytosis pathway. Furthermore, additional experiments showed that gp96, a β-1-4 linked N-acetyl glucosamine containing membrane glycoprotein, involved in the transcytosis of bacteria and toxins in epithelial and endothelial barriers, can serve as a receptor for BAAV transcytosis.

## Results

### BAAV Binds Chitotriose When Screened on a Glycan Microarray

**Figure 1:**
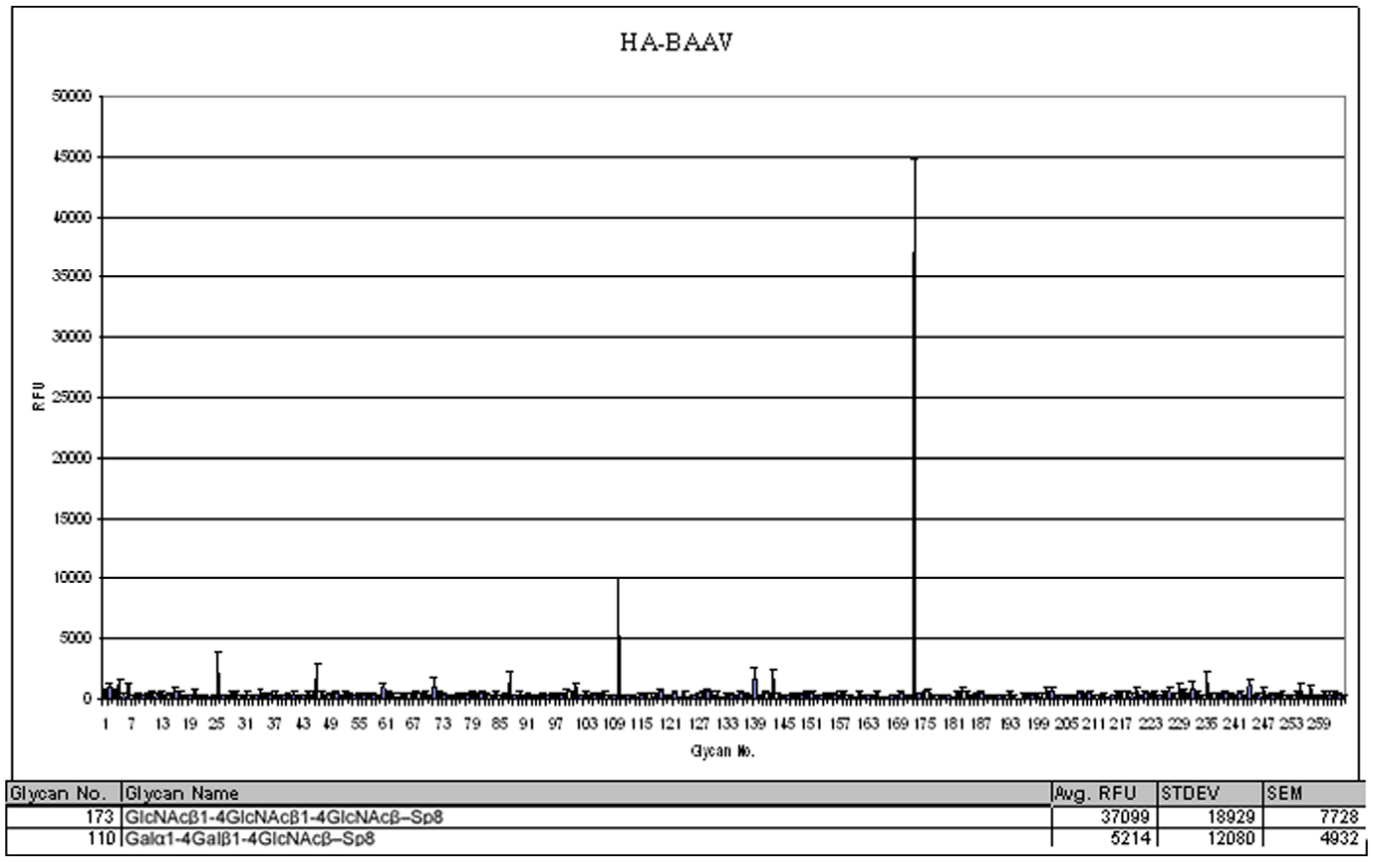
BAAV glycan binding on a glycan microarray. A printed glycan array slide containing 264 glycans (PA V2) was screened to identify stable BAAV carbohydrate interactions. The plot shows the average relative fluorescence (RFU) with the standard error measurement (SEM) for each glycan versus glycan number. The values for the top two hits (#173, GlcNAcβ1-4GlcNAcβ1-4GlcNAcβ–Sp8 or chitotriose, and # 110 Galα1-4Galβ1-4GlcNAcβ–Sp8 or the P1 blood group antigen) are listed below the figure.

In order to better define the carbohydrate interactions that are necessary for BAAV's life cycle we probed a glycan microarray composed of carbohydrates commonly found on the cell surface. The array is available through the Consortium for Functional Glycomics (CFG) and currently contains over 400 sialyated and nonsialyated glycans with different linkages and modifications and has been previously useful in characterizing AAV virus particle:carbohydrate interactions [20]. Purified BAAV vector genetically tagged with an HA epitope after amino acid 137 of VP1 and within the VP2 orf, a modification which has previously been shown not to effect the transduction activity of AAV2 [21], [22], was used to probe the printed slide (PA V2 http://www.functionalglycomics.org/static/consortium/resources/resourcecoreh8.shtml). The bound virus was detected using an FITC conjugated anti-HA antibody. The full summary of the microarray data is available at NCBI accession number GSE17443. The highest interaction was seen with glycan #173, GlcNAcβ1-4GlcNAcβ1-4GlcNAcβ–Sp8 or chitotriose (Fig. 1). Although significantly lower, BAAV vectors also showed weak affinity for glycan #110 Galα1-4Galβ1-4GlcNAcβ–Sp8 or the P1 blood group antigen. The same array probed with an HA tagged rAAV4 particles under the same conditions did not show these interactions (data not shown).

### Chitotriose Inhibits BAAV Transcytosis but Not Transduction

Chitotriose is a trimer of β-1-4-linked N-acetyl glucosamine units and can competitively inhibit lysozyme C [23]. Multimers of N-acetyl glucosamine are found as a post-translational modification on proteins and competition experiments with chitotriose can block the transcytosis of pathogens [24], [25].

**Figure 2:**
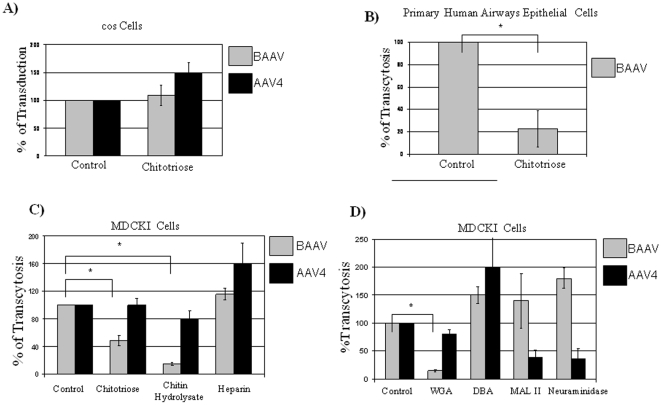
Effects of chitotriose, chitin hydrolysate, and lectins on BAAV transduction and transcytosis. (A) BAAV transduction is not affected by chitotriose. Cos cells plated in 96 well dishes were transduced in serial dilution with either BAAV or AAV4 vector encoding a CMV-eGFP expression cassette in either the presence or absence of 20mg/ml chitotriose. (B, C) Chitotriose and chitin hydrolysate inhibits BAAV transcytosis. DNAse resistant particles (DRP) of recombinant BAAV or AAV4, are applied on the upper (apical) side of the monolayer of cells grown on membrane insert filters (Transwell) either alone or in the presence of chitotriose (20mg/ml), chitin hydrolysate (125µg/ml), or heparin (20µg/ml). Three hours post incubation, viral DNA was extracted from the basal side medium and quantified by QPCR and expressed as a percent of the amount of vector in the basal media in the absence of any inhibitors. (B) HAE cells (C) MDCKI cells. N = 4. (D) Lectins inhibit BAAV or AAV4 transcytosis. Viruses were incubated with cells in presence of wheat germ agglutinin WGA 30µg/ml, dolichos biflorus agglutinin (DBA) 30µg/ml, maackia amurensis lectin II (MALII) 30µg/ml or neuraminidase 0.2µg/ml. Three hours post incubation, viral DNA was extracted and quantified and expressed as a percent of the amount of vector in the basal media in the absence of any inhibitors. * P<0.05.

In order to understand the role of the chitotriose carbohydrate in the life cycle of BAAV, cos cell transduction by BAAV vector virions encoding GFP was analyzed following pre-incubation with this glycan. The addition of chitotriose did not inhibit BAAV transduction of the cos cells, but rather slightly increased (10%) transduction activity (Fig. 2A). AAV4 transduction was also increased (50%) in the presence of chitotriose (Fig. 2A). Primary human airway epithelia (HAE) and MDCK1 cells have been shown to be permissive to both BAAV and AAV4 transcytosis (approx. 0.2–1% of total input vector will transcytose through the cells in 3 hrs [11]) and form a stable polarized monolayer that develops a trans epithelial resistance (TER) [11]. In contrast to the transduction assay, the addition of chitotriose blocked the transcytosis of BAAV when the virus was pre-incubated with the glycan prior to application to the apical surface of the cells. The addition of 20mg/ml decreased BAAV transcytosis activity almost 80% on HAE, and by 50% on MDCKI (Fig. 2B and C). In contrast, chitotriose did not significantly affect AAV4 transcytosis (Fig. 2C). In addition to chitotriose, chitin hydrolysate, a crude mixture of chito-oligomers that has previously been used to block transcytosis of Ecoli K1, a chitotriose dependent pathogen, was also tested. Titration experiments on MDCKI cells indicated that TER was unchanged at concentrations of chitin hydrolysate up to 250µg/ml (data not shown). Addition of 125µg/ml chitin hydrolysate inhibited ∼80% of the BAAV transcytosis but had no significant effect on the transcytosis of AAV4 (Fig. 2C). Moreover, addition of heparin sulfate, another charged glycan, resulted in marginal to moderate (∼10% and 60%) increases in the BAAV and AAV4 transcytosis activity, respectively (Fig. 2C).

The core structure of chitotriose is a β-1-4-linked N-acetyl glucosamine, which is reportedly bound by several lectins, including wheat germ agglutinin (WGA) [26]. In competition experiments, preincubation of MDCKI cells with 30µg/ml of WGA inhibited 85% of BAAV transcytosis activity but had no significant effect on AAV4 transcytosis (Fig 2D). Higher concentrations of lectins resulted in a decrease in TER (data not shown). A similar level of inhibition was also observed at 10µg/ml (data not shown). In contrast, dolichos biflorus agglutinin (DBA), which binds N-acetylgalactosamine, had the opposite effect on both viruses and resulted in ∼50% and 100% increase in transcytosis for BAAV and AAV4, respectively (Fig. 2D) [27]. Sialic acid linked to gangliosides is important in the transduction of BAAV [7] and for AAV4, O-linked sialic acid has been shown to be important for its transduction [7]. To test the role of sialic acid, and therefore gangliosides in BAAV transcytosis, MDCKI cells were either preincubated with the maackia amurensis lectin II (MALII), which binds specifically to sialic acid, or treated with neuraminidase, which enzymatically removes cell surface sialic acid, prior to the addition of vector (Fig. 2D). In both cases no loss of transcytosis was detected for BAAV, which suggests that unlike transduction, sialic acid and therefore gangliosides, do not have a role in BAAV transcytosis. In contrast AAV4 transcytosis was inhibited by ∼60% following both treatments (Fig. 2D). Taken together these experiments strongly suggest that the interaction of BAAV with chitotriose and chito-oligomers are important for BAAV transcytosis and that these interactions are virus specific. Furthermore, inhibition of BAAV transcytosis on both HAE and MDCKI epithelial barrier models suggests the vector may use the same transcytosis pathway on both cell types.

### Chitotriose Blocks BAAV Tannic Acid Mediated Transduction and Virus Entry

**Figure 3:**
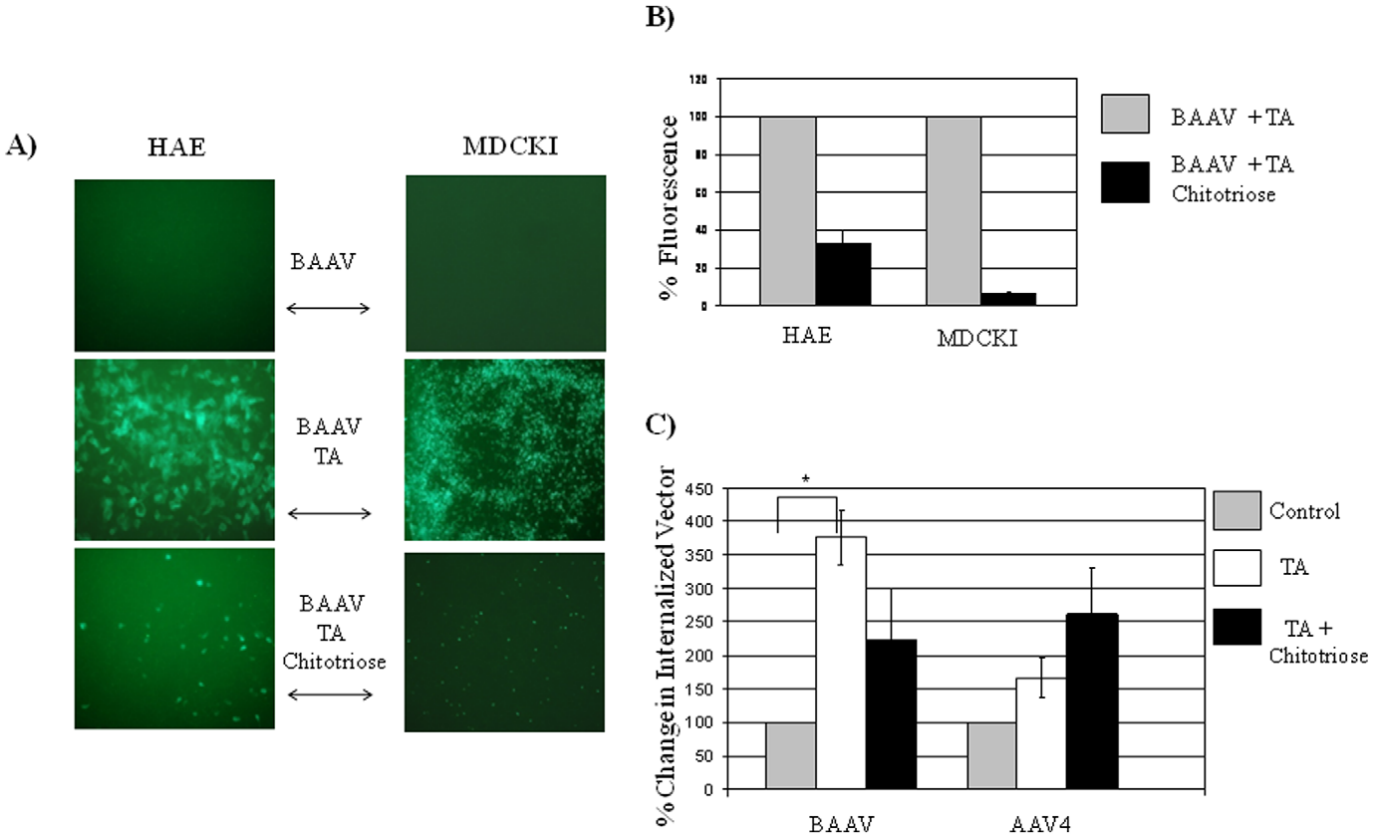
Chitotriose inhibits BAAV TA mediated transduction and cellular virus entry. (A) Tannic acid was added to the basal medium of HAE or MDCKI cells while vector was added either alone or in the presence of chitotriose (20mg/ml) to the apical surface. Transduction of cells treated with BAAV, BAAV and TA or BAAV, TA and chitotriose was observed 48 h post-vector addition by fluorescence microscopy. (B) Fluorescence comparison. Value of cells treated with BAAV TA +/− chitotriose was calculated after subtracting fluorescence background from BAAV only treated cells and expressed as a percent of fluorescence of the BAAV TA treated cells. (C) BAAV internalization in presence of chitotriose. MDCKI cells were incubated with BAAV or AAV4 in presence of tannic acid with or without chitotriose as above. Four hour post administration, cells were washed extensively with PBS and non-internalized bound particles were removed by incubating the cells with trypsin and protease K. Total DNA was isolated from the cells by phenol/chloroform extraction and viral genomes quantified by qPCR using primers specific for the promoter region. * P<0.05.

Our findings suggested that BAAV, like *E. coli* K1, requires an interaction with β-1-4 linked N-acetyl glucosamine for transcytosis to occur. To confirm that the chitotriose inhibition of transcytosis is due to its effect on cell entry and not due to the redirection of the vector to alternative pathways, we tested the effect of chitotriose on BAAV tannic acid (TA) mediated transduction (Fig. 3A). Previously we had demonstrated that treatment of the basolateral surface of cells grown on a Transwell with low concentrations of TA could block vector egress from the cell [11]. Furthermore, the internalized particles were redirected to the nucleus resulting in transduction [11]. In agreement with our earlier observation, the addition of chitotriose was able to block TA mediated transduction of BAAV on HAE and MDCKI cells (Fig. 3A and B). 48 hrs post vector addition, transduction with BAAV/chitotriose vectors encoding GFP on TA treated HAE or MDCKI cells decreased 3 and 15 fold respectively compared with that of TA controls (Fig. 3A and B, BAAV TA chitotriose versus BAAV TA). As expected, BAAV alone was not able to transduce the cells (Fig. 3A, BAAV).

These observations suggest that the BAAV vector particles are likely entering the cell via transcytosis and that chitotriose blocks this entry mechanism. Transcytosis is a complex process and likely involves multiple interactions with cellular factors. In order to determine if chitotriose is affecting the entry of BAAV particles into the cell, qPCR was used to measure the number of internalized genomes in the TA treated cells either +/− the addition of chitotriose (Fig. 3C). After incubation with BAAV particles +/− chitotriose the cells were extensively washed and treated with proteases to remove BAAV particles on the cell surface followed by isolation of total DNA and quantification by qPCR. As previously demonstrated, by treating the cells first with TA to block the egress of the particles, apical administration of BAAV on MDCKI cells increased the level of vector in the cell by ∼3-fold compared with the control samples. However, the addition of chitotriose reduced the amount of internalized genomes by 40% compared with the TA only treated samples (Fig. 3C BAAV). AAV4 has transcytosis activity on MDCKI cells and like BAAV its transduction on these cells can be increase by treating the basolateral surface of these cells with TA. In agreement with our results with BAAV, detection of AAV4 genomes in the TA treated cells also increased (by ∼70–100%) compared with untreated control cells. However, this increase in internalized particles was not inhibited by addition of chitotriose but rather resulted in a further increase of ∼100% (Fig. 3C AAV4). Taken together these observations show that chitotriose inhibits BAAV transcytosis and TA transduction by impeding the entry phase of transcytosis and further confirms that the AAV4 transcytosis phenomenon is chitotriose independent.

### Identification of gp96 As a Receptor for BAAV Transcytosis

**Figure 4:**
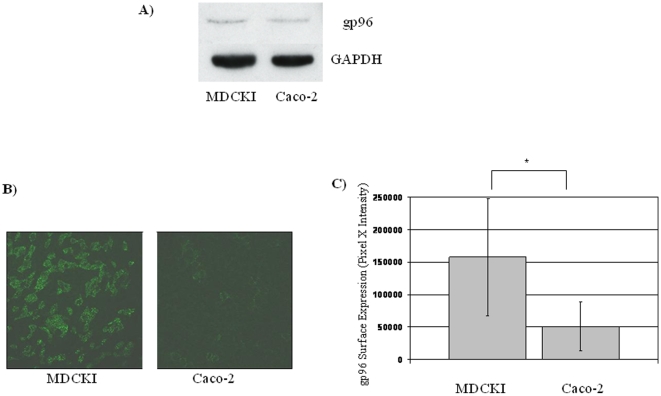
gp96 quantification and cell membrane distribution in BAAV transcytosis permissive (MDCKI) and non-permissive (Caco-2) cells. (A) gp96 western blot from whole cell lysates. GAPDH detection was used as a loading control. (B) Non-permeabilized cells were stained with anti-gp96 followed by Alexafluor 488 secondary antibody and analyzed by confocal immunofluorescence laser microscopy. (C) Fluorescence quantification of 10 random fields. * P<0.05.

**Figure 5:**
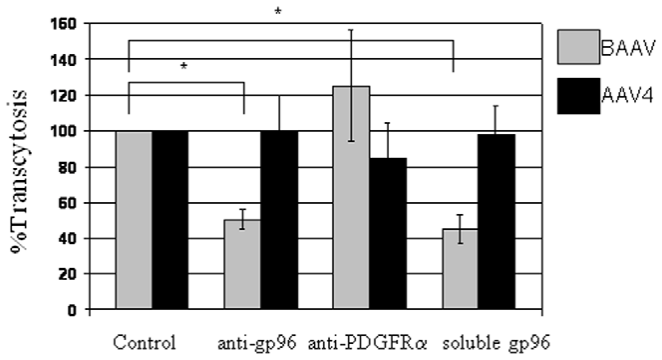
BAAV:gp96 competition experiments. Anti gp96, anti PDGFRα or soluble gp96 extra cellular domain, 25µg/ml, were placed on the apical surface of MDCKI cells grown on Transwell, prior to the addition of BAAV or AAV4. Transcytosis activity was measured by QPCR after 4 h. Values are expressed relative to the control (BAAV or AAV4 alone). * P<0.05.

The transcytosis pathway followed by BAAV is very similar to that reported for *E. coli* K1. Both are inhibited by WGA lectin, chitotriose, chitin hydrolysate, treatment of cells with filipin, and it can occur on in vitro cultures of blood brain barrier (BBB) endothelial cells [11], [25]. Extensive research on *E. coli* K1 transcytosis has identified a close homolog of gp96 as the protein receptor responsible for transcytosis across the BBB [24]. Recent reports suggest that gp96 can also function as a receptor for *Clostridium difficile* toxin A transcytosis, *Listeria monocytogenes* invasion and *Neisseria gonorrhoeae* cell attachment [28]–[30]. gp96 is a member of the heat shock protein family and localized to the endoplasmic reticulum where its chaperone function is critical for the proper folding of many substrates [31]. However, under appropriate conditions it also can be found on the apical membrane ([28], [30] and P.K. Srivastava personal communication). MDCKI and Caco-2 cells differ in BAAV transcytosis activity by 3–4-fold [11]. In order to determine if the cellular expression and localization of gp96 correlated with BAAV transcytosis activity in these cells, western blots analysis and cell immunofluorescence assays were performed. Western blots of whole cell lysates from MDCKI and Caco-2 showed a similar level of gp96 expression (Fig. 4A). However, confocal immunofluorescent plasma membrane imaging of cells with an anti-gp96 followed by labeled secondary antibody, showed a more uniform distribution of gp96 on permissive MDCKI cells compared with the more weakly permissive Caco-2 cells where it appeared more scattered and in clusters (Fig. 4B). Quantification of plasma membrane fluorescence for these cells showed a difference (∼3-fold higher) in pixel intensity representing the gp96 distribution in MDCKI cells compared with Caco-2 cells consistent with the overall difference in transcytosis activity (Fig. 4C). This observation suggests that gp96 may be involved in BAAV transcytosis. To investigate a direct role of gp96 in BAAV transcytosis, competition experiments using an anti-gp96 antibody or the soluble gp96 extracellular domain were performed. MDCKI cells grown on a Transwell were treated with either a polyclonal antibody to gp96 or PDGFRα (25 µg/ml), a receptor for AAV5, as a negative control, prior to the addition of BAAV or AAV4 vectors. Four hours post incubation with vector, transcytosis was measured. In contrast to treatment with the anti-PDGFRα antibody, transcytosis was decreased by 50% following treatment with the anti-gp96 antibody whereas that of AAV4 was unaffected (Fig. 5 control, anti-gp96, anti-PDGFRα). Furthermore, the addition of a soluble form of gp96 (25 µg/ml) had a similar effect on BAAV transcytosis but no significant effect on AAV4 (Fig. 5 control, soluble gp96). These observations point to the specificity of the gp96 recognition by BAAV and not AAV4.

**Figure 6:**
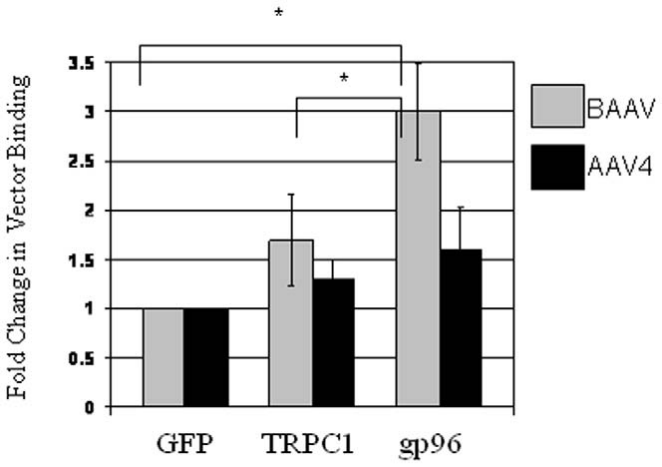
BAAV:gp96 immuno-precipitation. Anti-myc sepharose beads previously incubated with cell lysates either of GFP, or myc-tagged TRPC1 or gp96 transfected cells were then added with BAAV or AAV4 vector respectively containing the CMV-GFP expression cassette. After incubation at 4C° for 1h, beads were extensively washed and bound virus was quantified by QPCR. Bound vector was calculated as fold change in bound vector relative to vector bound to GFP beads alone. n = 3. * P<0.05.

The above data suggests a direct interaction between BAAV and gp96. Alternatively, BAAV could be following the same internalization pathway as gp96. To test for a direct interaction between BAAV and gp96, a myc tagged version of gp96 was expressed in 293T cells and purified using anti-myc affinity beads. The protein coated beads were then used in an immunoprecipitation experiment. The gp96 coated beads or control beads coated with either GFP or a myc-tagged version of the membrane protein TRPC1 were then incubated with purified BAAV or AAV4 viral particles. After extensive washing the bound virus was quantified by qPCR with primers specific for the reporter gene cassette. Compared with control GFP or TRPC1 beads, a 2–3fold increase in the amount of bound BAAV was detected when incubated with the gp96 coated beads (Fig. 6). In contrast, little difference in AAV4 binding to GFP, TRPC1 or gp96 coated beads was observed (Fig. 6). This pull down experiment indicates a direct interaction between BAAV and gp96.

## Discussion

The pathogenicity of a virus can be linked to its cell tropism and its ability to spread through tissue. Indeed, studies with other parvovirus such as MVM and CPV suggest that mutations that affect virus:cell interactions can alter pathogenicity, invasiveness, and host range [32], [33]. For many viruses, the first host defenses that must be overcome are the barrier epithelia that line the lung, gastrointestinal, or reproductive tract (for review see [4]). To overcome this obstacle, viral entry through normal signaling and trafficking pathways present in these cells represent an attractive target. A number of mechanisms exist in barrier epithelia for moving macromolecules from the apical to the basolateral surface. These include paracellular and passive pores routes, nonselective transcytosis in the fluid phase of vesicles, or selective receptor-mediated vesicle transcytosis [12]. Despite all these options, most pathogens are reported to penetrate barrier cell layers via a receptor-mediated pathway.

*Listeria monocytogenes* and *E. coli* K1 are examples of pathogens that use gp96 for transcytosis across barrier cell layers. *Neisseria gonorrhoeae* uses gp96 as an attachment receptor while *S. pneumoniae* translocation across a barrier layer via the poly(IgA) receptor [14], [15], [28], [30], [34].

Our previous work suggested that AAV transcytosis occurs via a receptor mediated pathway that is distinct from the pathway used for infection [11]. In this study glycan microarray analysis of BAAV carbohydrate interactions identified an interaction between BAAV and chitotriose. Competition assays with this carbohydrate showed that it did not block transduction but did inhibit transcytosis in vitro. Additionally, western blots, immunoprecipitation, and competition experiments were used to link BAAV's ability to bind chitotriose (a trimer of β-1-4-linked N-acetyl glucosamine) with the post-translationally modified glycoprotein gp96, suggesting that this protein can serve as a receptor for BAAV transcytosis. The use of AAV4 as a “negative” control throughout these studies demonstrated the serotype specificity of the chitotriose and gp96 interactions with BAAV.

Glycan microarrays are powerful tools for identifying interactions with carbohydrates. However, as with any array platform it is limited to what is printed on the array. We have previously reported sialic acid containing glycoshingolipids are necessary for BAAV transduction [7]. Although the chip contains a number of sialyated carbohydrates and some gangliosides on the array, there is no binding with these carbohydrates, suggesting either an interaction with distinct gangliosides not on the array is required for transduction or an interaction with other molecules is required to stabilize the interaction with gangliosides. Detection of other carbohydrate interactions that are critical to BAAV transduction will require alternative methods of detection or more extended versions of glycan microarrays.

Like transduction, AAV transcytosis could be a complex process. Although our data does show a direct interaction between gp96 and BAAV, the lack of complete inhibition in the presence of the anti-gp96 antibody and soluble gp96 suggests that other membrane components not present with the purified recombinant gp96 used in the immunoprecipitation experiment may contribute to the interaction. In addition other factors may also be required for internalization. A multistep interaction process during cellular entry has been reported for other AAVs. For example, cellular transduction by AAV2 requires an interaction with a primary heparan sulfate proteoglycan receptor as well as several co-receptors, the integrins αVβ5 and α5β1, fibroblast growth factor receptor and hepatocyte growth factor receptor, for cellular recognition and internalization [35]–[38].

To date we have identified transcytosis activity in at least 3 AAV serotypes (AAV4, AAV5 and BAAV) and this phenomenon has been suggested to occur in AAV8 and AAV9 [11], [16]–[19]. Two outstanding questions are (I) whether or not this is a process that is common to all AAVs and (II) whether or not the other AAVs also use gp96 for this cellular transport process that is independent of the transduction pathway. Given the sequence divergence, utilization of distinct receptors for transduction, and our previous work describing distinct transcytosis profiles on the different AAV serotypes, it is possible that each virus will use a distinct transcytosis pathway if they have transcytosis activity. Indeed the data presented for AAV4 in this paper suggests that sialic acid is an important carbohydrate in its transcytosis pathway. Analysis of the chitotriose/gp96 binding site on BAAV will lead to a better understanding of the transcytosis activity in the lifecycle of the AAVs and the potential to provide information that could be exploited for new applications of AAV gene transfer vectors, such as the engineering of vectors which could navigate the blood brain barrier.

## Materials and Methods

### Cell Cultures, Vector Construction, Preparation, and Quantification

293T (human kidney) and cos cells were maintained in Dulbecco's modified Eagle's medium supplemented with 10% fetal bovine serum (FBS). The media contained 2 mM L-glutamine, 100 U of penicillin/ml, and 0.1 mg of streptomycin/ml. Cells were maintained at 37°C under a 5% CO_2_ humidified atmosphere.

Recombinant BAAV, BAAV-HA, AAV4-HA, and AAV4 viruses expressing eGFP were produced using a four-plasmid procedure as previously described [8]. Briefly, semiconfluent 293T cells were transfected by calcium phosphate with four plasmids: an adenovirus helper plasmid (pAd12) containing the VA RNA and coding for the E2 and E4 proteins; two AAV helper plasmids containing the AAV2 *rep* and AAV specific *cap* genes, respectively, and a vector plasmid containing AAV2 inverted terminal repeats flanking an eGFP expression cassette. Forty-eight hours post-transfection the cells were harvested by scraping into TD buffer (140 mM NaCl, 5 mM KCl, 0.7 mM K2 HPO_4_, 25 mM Tris–HCl {pH 7.4}) and the cell pellet was collected by low-speed centrifugation. Cells were lysed in TD buffer by three cycles of freeze–thaw. The clarified lysate (obtained by further low-speed centrifugation) was treated with 0.5% deoxycolic acid (DOC) and 100 U/ml DNase (Benzonase) for 30 min at 37°C. Then the virus was purified using CsCl gradients. Particle titers were determined by qPCR. Amplification was detected using an ABI 7700 sequence detector (ABI). Specific primers for CMV were designed by using the Primer Express program (ABI): CMV forward 5′-CATCTACGTATTAGTCATCGCTATTACCAT-3′, CMV reverse 5′-TGGAAATCCCCGTGAGTCA-3′. Following denaturation at 96°C for 10 min, cycling conditions were 96°C for 15 s, 60°C for 1 min for 40 cycles. The viral DNA in each sample was quantified by comparing the fluorescence profiles with a set of DNA standards. The rAAV particle titers were 1–5×10^12^ DNAse resistant particles (DRP) per ml.

The HA epitope tag (YPYDVPDYA) was added to BAAV after amino acid 137 in the VP1 open reading frame to create BAAV-HA using a site directed mutagenesis kit (Stratagene). The following primers were used: Forward 5′ gagacgccggataaaacgtacccatacgacgttccagactacgcagcgcctgcggcaaaaaagaggcc 3′ Reverse 5′ggcctcttttttgccgcaggcgctgcgtagtctggaacgtcgtatgggtacgttttatccggcgtctc 3′. The presence of the tag into the clone was verified by sequencing. This modification provided an antigenic epitope for BAAV detection in glycan microarray screening used to identify carbohydrates that bind this virus as described below.

### Glycan Microarray

BAAV-HA particles were produced as described above and concentrated to a titer of ∼1×10^14^ vg/ml. The concentrated virus was then dialyzed into a Tris buffer (Tris pH7.5 w/150 mM NaCl) and used to probe a printed glycan array (PA V2) following procedures developed by cores D and H of the Consortium for Functional Glycomics (CFG; an NIH National Institute of General Medical Sciences initiative (http://www.functionalglycomics.org/static/consortium/resources/resourcecoreh.shtml) for identifying specific carbohydrate binding partners for protein and viral lectins [39]. Briefly, a printed slide containing 264 glycans was incubated with AAV particles (at 200 µg/ml), and overlaid with a FITC conjugated anti-HA tag (Molecular Probes Invitrogen) at 5µg/ml. The fluorescence intensity was detected using a ScanArray 5000 (Perkin-Elmer Inc.) confocal scanner. The image was analyzed using the IMAGENE image analysis software (Bio- Discovery, El Segundo, CA). The data were plotted using the Microsoft EXCEL software. NCBI accession number GSE17443 is MIAME compliant.

### Epithelial Barrier Cell Models

Caco-2, and MDCKI cells were grown at 37°C, in 5% CO_2_ in DMEM containing 10% FBS, 1% Pen/Strep (Biosource, CA, USA). Human primary airway epithelia cells (HAE) were purchased from Lonza (MD, USA) or Promocell (Heidelberg, Germany) and were grown at 37°C, 5% CO_2_ in medium supplied by the cell provider. HAE cells were expanded and differentiation was induced as previously described [40] the generation of cell barrier models for transcytotic assay were carried out as previously described [11]. Briefly, all cell types were allowed to establish monolayers on 0.4-µ pore size polycarbonate filters in 6 or 12 mm Transwell chambers (Costar, MA, USA) and the integrity of cell monolayers, polarization, and formation of tight junctions were tested by measuring the Transepithelial Electrical Resistance (TER) using a volt/ohm meter (Millicel; Millipore, MA, USA) in an electrode chamber (EVOV; WPI, FL, USA). Only filters of cell monolayers that displayed the required TER were used for the AAV transcytosis assay, i.e. MDCKI, 2000 Ω/cm^2^; Caco-2 200–300 Ω/cm^2^, HAE ∼1000 Ω/cm^2^, as previously published [11], [12], [40,]. As previously reported, no movement of particles across the membrane was detected on empty Transwells confirming the seal of the chamber [11]. All experiments with HAE cells were performed on air–liquid interface cultures.

### BAAV Transduction Assay

cos cells were plated in 96 well dishes and incubated, in serial dilution, with either BAAV or rAAV4 vector encoding a CMV-eGFP expression cassette in either the presence or absence of 20mg/ml chitotriose (Sigma). Three days post incubation positive cells were counted.

### BAAV Transcytosis Assays

2×10^8^ DRP of BAAV or rAAV4, suspended in 50 µl of DMEM, were placed on the upper (apical) side of the monolayer of cells grown on 6 mm membrane insert filters (Transwell, Costar) either alone or in the presence of chitotriose or heparin. With air–liquid interface HAE cultures, 20 µl of medium was added to the apical surface. 12 mm insert filters were the membrane surface is 3 fold larger, medium and virus were increased, accordingly. After 3 h of incubation, the medium in the lower (basal) side of the Transwell was collected and tested for the presence of transcytosed rAAV DNA. Viral DNA was extracted from 200 µl of medium from the basolateral side of the Transwell using the DNeasy Mini Spin Column Kit (Qiagen, CA, USA) and quantified by qPCR as previously described. In tannic acid (TA) mediated transduction experiments medium containing 0.5% TA (Polysciences, PA, USA) was placed on the basolateral side of the Transwell inserts while BAAV was placed on the apical side with or without chitotriose. Transduction was observed 48 h post-vector addition by fluorescence microscopy. In carbohydrate competition experiments, chitotriose, chitin hidrolysate (Vectorlabs), heparin (Sigma) were incubated with BAAV or AAV4 10 min at 4°C prior the addition to the apical side of the cells. In competition experiments, lectins (Vectolabs), neuraminidase (Prozyme), anti-gp96 (Stressgen) or anti-PDGFRα (Santa Cruz) were added on the apical side of MDCKI cells 10 min prior to the addition of BAAV or AAV4. Whereas, soluble gp96 receptor (Stressgen) was incubated for 10 min with virus prior to cell incubation. Three hrs post addition transcytosed virus was quantified as described above.

### BAAV Internalization

Basolaterally TA treated MDCKI cells were incubated with either BAAV or AAV4 with or without chitotriose as described above. Four h post administration, cells were washed extensively with PBS and non-internalized bound particles were removed by incubating the cells with 0.05% trypsin-EDTA (Gibco) and 0.4 mg/ml protease K (Qiagen) until cells were detached from each other and from Transwell filters. Cells were washed three times with PBS, total DNA was isolated from the cells by phenol/chloroform extraction, and viral genomes quantified by qPCR using CMV primers.

### Western Blot Analysis for gp96

5×10^5^ MDCKI or Caco-2 cells grown on Transwell were lysed with 2% SDS containing 0.25 U benzonase (Sigma) and 1∶100 dilution of protease cocktail inhibitors (Sigma). Cell lysate was centrifuged at 11000g for 2 min to remove cell debris, aliquoted, and stored at −20 C. 50µg/lane of cell lysate was run on a 12% Bis-Tris gel (Invitrogen) and transferred using a semi-dry apparatus (Biorad, Richmond CA) to nitrocellulose membrane for 1 h at 15 volts. The membrane was blocked for 30 min in 5% milk/TBST (Tris Buffered Saline with 0.05% Tween20), then incubated with anti-gp96 (Santa Cruz) (1∶100 dilution as per manufacturer's protocol) in 5% milk/TBST followed by incubation with anti-mouse secondary antibody (1∶5000 dilution, Amersham) in 5% milk/TBST. All washes were carried out in TBST. The bands were visualized using ECL Chemiluminescent Substrate Reagent (Amersham). GAPDH detection was used as a loading control.

### gp96 Cell Membrane Distribution

MDCKI or Caco-2 cells grown on Transwell were fixed in 2% formaldehyde/PBS for 10–12 min. Non-permeabilized cells were probed with anti-gp96 (Santa Cruz) in 10% FBS in PBS/0.02% sodium azide for 1 h followed by a goat anti-rabbit Alexafluor 488 (Molecular Probes, Invitrogen) secondary antibody in 10% FBS in PBS/0.02% sodium azide for 1 h and analyzed by confocal immunofluorescence laser microscopy. Fluorescence quantification of 10 random fields was obtained using Volocity software (Improvision, Waltham, MA, USA).

### gp96/BAAV Immunoprecipitation

293 cells were transfected with gp96-myc (a gift from Christopher Nicchitta), TRPC1-myc (a gift from Biman Paria), or GFP expression plasmids using Lipofectamine 2000 (Invitrogen) according to the vendor's protocol. Forty-eight hrs post transfection, cells expressing gp96, TRCP1, or GFP were lysate in M-Per reagent (Pierce, Rockford, IL) and immunoprecipitated (IP) using the Mammalian c-Myc Tag IP/Co-IP kit (Pierce, Rockford, IL). Anti-myc agarose beads previously incubated with cell lysates either of GFP, TRPC1 or gp96 myc tagged transfected plasmids were then mixed with 10^8^ DRP of BAAV or AAV4 vector containing the CMV-GFP expression cassette. After incubation at 4C° for 1 h, the beads were extensively washed with TBS-T buffer in spin columns. The DNA of bound vector was extracted by phenol/chloroform and quantified by qPCR. Vector bound to the anti-myc beads was calculated as fold change in bound vector relative to vector bound to GFP beads alone. These experiments were done in triplicate (n = 3).

### Statistics

All data are expressed as means +/− standard error measurement (SEM). Unpaired Student's *t*-test was used to assess the significant difference between groups. P values <0.05 were considered significant.
